# Adaptation and Validation of the Spanish Version of the Instrument to Evaluate Nurses’ Attitudes Toward Communication With the Patient for Nursing Students

**DOI:** 10.3389/fpsyg.2021.736809

**Published:** 2021-11-25

**Authors:** María del Carmen Giménez-Espert, Sandra Maldonado, Daniel Pinazo, Vicente Prado-Gascó

**Affiliations:** ^1^Nursing Department, Faculty of Nursing and Chiropody, University of Valencia, Valencia, Spain; ^2^Nursing Department of the School of Health Sciences, Human Services and Nursing, Lehman College, CUNY New York, Bronx, NY, United States; ^3^Developmental, Educational, Social and Methodological Psychology Department - Social Psychology, Universitat Jaume I, Castellón de la Plana, Spain; ^4^Social Psychology Department, Faculty of Psychology, University of Valencia, Valencia, Spain

**Keywords:** attitude, communication, construct validation, nursing students, psychometric properties

## Abstract

Communication is essential to the quality of care and patient satisfaction. It has been linked to positive patient outcomes, increased engagement, improved health outcomes, and safe practices. Given these benefits and the association between attitudes and behaviors, as behaviors can be predicted by studying attitudes, assessing attitudes of nursing students toward patient communication is critical for future nursing professionals. For this purpose, the main aim of this study was to adapt and validate an instrument to measure nurses’ attitudes toward communication (ACO) for nursing students. The ACO with patients was analyzed. Then, differences in the dimensions of the instrument (ACO) for nursing students according to an academic course and the correlations were calculated. A cross-sectional study was carried out in a convenience sample of 1,417 nursing students from five universities in the Valencian Community (Spain) during the 2018/2019 academic year and 83.8% (1,187) were women. The reliability was analyzed by using Cronbach’s alpha and composite reliability (CR). Analysis of construct validity was performed with exploratory factor analysis (EFA) and confirmatory factor analysis (CFA). The instrument adapted from nurses to nursing students was composed of 25 items grouped in three dimensions: affective, cognitive, and behavioral. The psychometric properties suggested that the instrument ACO for nursing students was reliable and valid. The ACO of nursing students was positive with high levels in cognitive and behavioral dimensions, while scores were worst in the affective component. The second-year nursing students showed more positive attitudes in the affective dimension, while in the cognitive and behavioral dimensions, the most positive attitudes were found in the first year. In the correlations, the behavioral and cognitive dimensions showed a significant, positive, and very high correlation. These findings should be considered in developing academic plans to improve the effectiveness of the communication education process of the students to increase the quality of patient care and well-being of nursing students.

## Introduction

The importance of communication is reflected in the theories and models of nursing that support the professional practice; in this sense, the Theory of Human Caring proposed by Jean Watson (Watson, 2018) highlights the importance of open communication with the patient and his/her family in the care process. In the care process according to Watson, the nurse should interact with “the person” rather than with “the patient,” understand their beliefs, emotions, feelings, and fears, without forgetting their individuality and knowledge (Foronda et al., 2016; Wei and Watson, 2019). Communication is vital in all the areas of nursing care: prevention, treatment, rehabilitation, education, and health promotion (Kourkouta and Papathanasiou, 2014); it is a process by which information is exchanged and shared during interventions performed with the patient (Hannawa et al., 2015). In short, it is the essence of the relationship with the patient to create a positive interpersonal relationship, exchange information and make appropriate decisions related to treatment and care (Grassi et al., 2015), and sharing of ideas, thoughts, feelings, and needs with another person (Xie et al., 2013). All the nurses are expected to be competent in communication (Mullan and Kothe, 2010), i.e., able to communicate effectively with patients, their families, and other members of the healthcare team (Claramita et al., 2016).

Nurse-patient communication is ultimately an interpersonal communication in which an exchange of information is carried out between the patient and the professional from a comprehensive and holistic view of the person, which allows knowing their real needs and, therefore, to establish a therapeutic relationship. Therapeutic communication between nurse and patient is considered the basis of nursing care (Abdolrahimi et al., 2017a); it is patient-centered and involves physiological, psychological, environmental, and spiritual aspects of the patient (Peplau, 1991). It is based on understanding and addressing the situation of the patient, including relevant life circumstances, beliefs, perspectives, concerns, and needs in order to plan appropriate patient care (Cusatis et al., 2020). Therapeutic communication is essential to the quality of care and satisfaction of the patient (Finke et al., 2008; Kourkouta and Papathanasiou, 2014; Finney Rutten et al., 2015; Banerjee et al., 2016; Gillett et al., 2016; Howick et al., 2018). It has been linked to positive patient outcomes, increased engagement, improved health outcomes (Kitson et al., 2014; Burgener, 2020), safe practices (Lin et al., 2017), and decrease the emotional burden on both the nurses and families (Charlton et al., 2008; Wittenberg et al., 2017). From the perspective of patients, it enhances the trusting relationship that can be built with nurses and facilitates decision-making (Rosemond et al., 2017). In addition, effective communication among multidisciplinary team members is critical to the effectiveness of healthcare teams and can be related to the quality of care and job satisfaction of the nurses (Gausvik et al., 2015). Communication errors can increase the incidence of adverse events and cause various harm to patients (Li et al., 2019). World Health Organization (2017) identifies communication as an essential tool of patient safety culture and a cause of delay in treatment, medication errors, and incorrect procedures (The Joint Commission, 2016).

Given the benefits of therapeutic communication for good care, for safe, and quality practice (Boschma et al., 2010; Finney Rutten et al., 2015), it is critical to address these issues in nursing students as future nursing professionals (Grant and Jenkins, 2014). Literature shows that communication can be a challenge for nurses and nursing students (Suzuki et al., 2014), as studies assess communication skills showing that these skills are poor among nurses (Hemsley et al., 2012; Shorey et al., 2018). Other studies have shown that communication skills are also deficient among nursing students such that they are a problem for their well-being (Satu et al., 2013). Students identified numerous barriers to effective communication, including reluctance to engage with patients or families, difficulty in initiating or sustaining conversation, feeling devalued, frightened, fearful, or anxious, and continuing to worry about their performance after the interaction is over (Beckstrand et al., 2012; Banerjee et al., 2016; Lin et al., 2017). Previous studies indicate that interventions to teach nurse-patient communication skills focus on more difficult clinical interactions (MacLean et al., 2017), mental health patients (Sarikoc et al., 2017), palliative care (Coyle et al., 2015), hence these are more studied areas. However, communication skills in general care settings with patients are equally important (Chan, 2014).

Therapeutic communication is based on the knowledge, attitudes, and skills of the patient and nurse that lead to patient understanding and participation (Abdolrahimi et al., 2017b). Despite this, most studies have focused on communication skills, communication knowledge, and medical students (Epstein et al., 2010; Škodová et al., 2018). There is not a comprehensive assessment of attitudes in the communication of nursing students before they are exposed to real human interactions during their clinical practice (Foronda et al., 2016). Therefore, studying attitude toward communication (ACO) in nursing students is important based on the “Theory of Reasoned Action” (Ajzen and Fishbein, 1980) because of the relationship between attitudes and the behavior of individuals. According to this theory, a change in behavior can be induced by a change in the attitude of the person and can predict behaviors by studying attitudes (Ajzen, 1991). The behavioral component of attitudes is a manifestation of the underlying cognitive and affective components (Anvik et al., 2007). This aspect is very important in nursing students because assessment of ACO allows identification of negative attitudes and perception of communication is an unimportant part of effective healthcare, which could negatively influence the effectiveness of the educational process (Škodová et al., 2018) in the integration of communicative knowledge and skills in nursing students (Fukada, 2018). In addition, studying ACO within the care process helps to evaluate the interpersonal communication of the nurse with the patient (Chan, 2017) in order to be able to adapt strategies and their effectiveness (Grant and Jenkins, 2014) to increase the quality of patient care and well-being of nursing students (Satu et al., 2013). Considering the importance of ACO in nursing students and there are no instruments for its assessment, it would be important to develop validated instruments to evaluate ACO with the patient (MacLean et al., 2017; Levett-Jones et al., 2019).

The literature provides scarce studies on the reliability and validity of the instruments used (Grant and Jenkins, 2014; Gutiérrez-Puertas et al., 2020) on ACO skills in nursing students; the Communication Skills Attitude Scale (CSAS) (Škodová et al., 2018) was originally developed to measure the ACO skills in medical students (Rees et al., 2002). Others measure communication competencies of nursing students such as the Interpersonal Communication Assessment Scale (ICAS) (Klakovich and dela Cruz, 2006) and the ACO of the nurses with patients (Giménez-Espert and Prado-Gascó, 2018), with adequate psychometric properties. So, in this study, the main aim of an adaptation and validation of this instrument in nursing students was performed. Thus, the ACO with patients in a sample of nursing students was analyzed. Finally, differences in the dimensions of the instrument (ACO) for nursing students according to an academic course and the correlations were calculated.

## Materials and Methods

### Participants and Study Settings

A cross-sectional study was carried out in a convenience sample of 1,417 nursing students from five universities in the Valencian Community (Spain). The nursing degree in Spain includes four full-time academic years with 240 European Credit Transfer System (ECTS) at 60 ECTS per year (1 credit representing between 25 and 30 h of student work). After completing the program, the student obtains a degree in nursing and can practice in Spain and in the European Union countries.

### Data Collection

The inclusion criteria were students enrolled in the nursing degree of the universities participating who gave their consent for participation after receiving information about this study. The anonymity and confidentiality of the information provided were indicated. The self-report instrument was completed in the classroom by the participants, which lasted around 10 min. The data collection phase was developed during the 2018/2019 academic year.

### Instrument

Attitudes Toward Communication of Nurses with the patient (ACO) (Giménez-Espert and Prado-Gascó, 2018) to measure the ACO of the nurses with the patient (intellectual property registered at the University of Valencia on 08/04/2019, registration number: UV-MET-201917R). The instrument is based on Rosenberg and Hovland’s (1960) three-dimensional model of attitude: affective, cognitive, and behavioral. The attitude components were related to the most important communicative moments of the hospitalization process (Duhamel and Talbot, 2004): admission, procedure, and discharge. The three communicative moments were related to the nursing interventions according to the Nursing Interventions Classification (NIC) (Bulechek, 2009). Finally, from this classification, the following interventions with their corresponding activities were selected and related to the three components of the attitude: nursing care at admission (7,310), teaching: procedure/treatment (5,618), and discharge planning (7,370). The instrument was composed of 25 items, grouped in three dimensions: Affective, related to situations and to admission, procedure, and discharge of the patient that generate anxiety in nurses (12 items, Cronbach’s α = 0.95, e.g., *“I’m nervous when I inform the patient and/or family about how they can help in recovery”*); behavioral, related to what nurses usually do with respect to the patient and/or family member regarding checking to understand information on admission, encouraging questions, aspects related to orientation in the unit (visiting hours, routines), reinforcing, facilitating and clarifying information to the patient to obtain informed consent, checking to understand information on discharge and its implementation, allowing time for questions, and how to collaborate during the procedure. It is that which we can observe and allows us to deduce the other two (9 items, Cronbach’s α = 0.92, e.g., *“I usually encourage the patient and/or family ask me when I provide information at the time of admission to the unit”)*; and cognitive, refers to the importance for nurses: orientation of the patient and/or family in the unit, information that can help in recovery, information on discharge care, and finally collaboration with other members of the healthcare team (4 items, Cronbach’s α = 0.85, e.g., *“It is necessary to inform the patient and/or family about how they can help in recovery”)*. A five-point Likert scale was used ranging from 1 = strongly disagree to 5 = strongly agree. High scores in all the dimensions correspond to positive ACO, except in the affective dimension where lower scores indicate more positive ACO, as it is an inverted dimension when considering the stress produced by communication. The original instrument showed adequate psychometric properties (Giménez-Espert and Prado-Gascó, 2018) and was modified for nursing students “Although I am a student now, I believe that when I practice as a nurse.” In this study, the student-adapted version of the instrument had acceptable reliability (Cronbach’s α = 0.84) (intellectual property registered at the University of Valencia on 30/07/2020, registration number: UV-MET-202044R).

### Design: Instrument Adaptation Process

The instrument adaptation process was performed in three stages:

*Stage 1: adaptation*

The international methodological standards for the adaptation of an instrument established by the International Test Commission (2017) were followed in the adaptation of the ACO of the nurses with the patient instrument for nursing students.

*Stage 2: content validity process*

The items were evaluated by a panel of experts and in a pilot sample of 100 nursing students to assess content validity, according to the accuracy, clarity, legibility, and relevance of each item of the instrument (Polit and Beck, 2008). The experts were five nurses with at least 10 years of clinical experience, training and research in the field (Polit and Beck, 2008). The content validity index (CVI) was calculated, and the criteria for inclusion of the item were that the CVI was larger than 0.80 (Lynn, 1986). Moreover, the comments of the nursing students and the experts were analyzed, and there were no items unclear or controversial. According to these results, the 25 items were maintained in the final version of the instrument.

*Stage 3: statistical analyses and psychometric properties*

The Statistical Package for the Social Sciences (SPSS) software (version 22), the EQS software (Structural Equation Modeling software, version 6.2) (Bentler, 2004), and the FACTOR software (Lorenzo-Seva and Ferrando, 2006) were used to perform the statistical analyses of this study. First, descriptive analysis of every item (mean and SD) and observations of the item-total correlation coefficients. The reliability was also analyzed by using Cronbach’s alpha and composite reliability (CR). Analysis of construct validity was performed with exploratory factor analysis (EFA) and confirmatory factor analysis (CFA). The convergent validity was assessed by using the results of the CFA, while for discriminant validity, the average variance extracted (AVE) test was used (Fornell and Larcker, 1981).

The adequacy of the proposed models by CFA was tested with the significance of χ^2^ (Bentler, 2004). The coefficients of the goodness of fit, the non-normed fit index (NNFI), the comparative fit index (CFI), and the incremental fit fix (IFI) values > 0.90 were considered as a good fit (McCallum and Austin, 2000). Finally, the root mean square error of approximation (RMSEA) was calculated, and it was required to be <0.08 to considered as a good fit (Browne and Cudeck, 1993).

The ANOVA was calculated to search for differences between academic year and the ACO dimensions for nursing students. The correlations were analyzed by using Pearson’s correlation coefficient.

## Results

### Sample Characteristics

The age of the participants ranges from 18 to 55 years (M = 21.80, *SD* = 5.34). According to the distribution by sex, 83.8% are women (*n* = 1,187) and 16.2% are men (*n* = 230). According to the course of study, the distribution observed is as follows: 29.6% first, 25% second, 23.8% third, and 21.6% fourth.

### Psychometric Evaluation of the Instrument

#### Analysis of the Items and Reliability

The attitudes toward communication instrument for nursing students is composed of 25 items distributed in three dimensions. Table 1 shows items grouped according to the dimension. In addition, the table collects for all the items such as the mean (M), SD, item-total correlation (rjx), and Cronbach’s alpha, if that item is eliminated (α-x). The ACO instrument for nursing students as a whole shows acceptable reliability (α = 0.84).

**TABLE 1:** Analysis of the attitudes toward communication (ACO) for nursing students items: Mean (M), SD, item-total correlation (r_*jx*_), and Cronbach’s alpha if it eliminates the element (α-x).

Complete instrument (α = 0.84)	*M*	*SD*	r_*jx*_	α -x
ACO 1	1.95	0.99	0.36	0.83
ACO 2	1.65	0.89	0.32	0.83
ACO 3	1.82	0.93	0.43	0.83
ACO 4	1.74	0.92	0.30	0.84
ACO 5	1.49	0.80	0.38	0.83
ACO 6	1.65	0.87	0.40	0.83
ACO 7	1.58	0.86	0.38	0.83
ACO 8	1.42	0.78	0.25	0.84
ACO 9	1.57	0.85	0.29	0.84
ACO 10	1.61	0.84	0.34	0.83
ACO 11	1.40	0.74	0.35	0.83
ACO 12	1.84	0.99	0.36	0.83
ACO 13	4.41	1.10	0.28	0.84
ACO 14	4.50	0.87	0.37	0.83
ACO 15	4.59	0.84	0.44	0.83
ACO 16	4.57	0.82	0.39	0.83
ACO 17	4.62	0.79	0.41	0.83
ACO 18	4.67	0.75	0.46	0.83
ACO 19	4.65	0.75	0.45	0.83
ACO 20	4.67	0.71	0.41	0.83
ACO 21	4.59	0.77	0.45	0.83
ACO 22	4.68	0.72	0.45	0.83
ACO 23	4.77	0.65	0.47	0.83
ACO 24	4.76	0.67	0.46	0.83
ACO 25	4.77	0.68	0.46	0.83

#### Construct Validity: Factor Analysis (Exploratory Factor Analysis and Confirmatory Factor Analysis)

An EFA was performed to examine how the items are distributed without any restriction. An EFA was performed following the process recommended by Lloret-Segura et al. (2014) by using the unweighted least squares method and normalized direct oblimin rotation. To determine the number of common factors in which the items are grouped, parallel analysis was used.

The EFA performed by using the FACTOR program (Lorenzo-Seva and Ferrando, 2006) with the 25 items of the ACO instrument for nursing students recommended the grouping of the items into two common factors. It was decided to check the fit of the factorial structure set on the three dimensions since it was difficult to interpret the two-factor factorial solution theoretically. After the application of the EFA fixed to three factors, it was not necessary to suppress any item since the saturations were higher than 0.40, maintaining the scale at 25 items. The fit of this solution was adequate with an RMSR value of 0.03 (<0.50; Harman, 1980) and a goodness of fit index (GFI) index of 0.99 (>0.95; Tanaka and Huba, 1989). The Kaiser–Meyer–Olkin (KMO) index of sampling adequacy also presented an optimal value (KMO = 0.93; 95% CI 0.918–0.920) and Bartlett’s test of sphericity was significant (χ^2^ = 13836.6; df = 300; *p* ≤ 0.001). The variance explained by the three factors was 78%.

Once the EFA was performed, CFA was completed to check the factor structure extracted by the EFA. This model presents adequate fit (χ^2^ = 2347.59; df = 272; *p* < 0.05; NNFI = 0.91; CFI = 0.92; IFI = 0.92; and RMSEA = 0.07 (95% CI = 0.076–0.082). Convergent validity showed that the items of the instrument were correlated with the latent variables (affective, behavioral, and cognitive). The loadings of each item were higher (>0.70; Anderson and Gerbing, 1988) except in items (ACO4 λ = 0.67, ACO8 λ = 0.66, ACO12 λ = 0.61, and ACO13 λ = 0.58). In every case, the T values for the variables ranged from 20.13 to 48.81 (*t* > 1.96) and were significant at the 0.05 level. The CR and the AVE for each dimension: affective CR = 0.98; AVE = 0.58, behavioral CR = 0.97; AVE = 0.53, and cognitive CR = 0.96, AVE = 0.82; CR (>0.70; Nunnally, 1978) and AVE (≥0.50; Fornell and Larcker, 1981; Table 2).

**TABLE 2:** Results of the CFA with factor loadings, composite reliability, and average variance extracted from ACO for nursing students.

Items		Factor loading	CR	AVE
	Dimension 1 affective		0.98	0.58
ACO 1		0.72		
ACO 2		0.77		
ACO 3		0.79		
ACO 4		0.67		
ACO 5		0.84		
ACO 6		0.83		
ACO 7		0.85		
ACO 8		0.66		
ACO 9		0.73		
ACO 10		0.74		
ACO 11		0.73		
ACO 12		0.61		
	Dimension 2 behavioral		0.97	0.53
ACO 13		0.58		
ACO 14		0.73		
ACO 15		0.85		
ACO 16		0.82		
ACO 17		0.85		
ACO 18		0.89		
ACO 19		0.85		
ACO 20		0.83		
ACO 21		0.82		
	Dimension 3 cognitive		0.96	0.82
ACO 22		0.87		
ACO 23		0.92		
ACO 24		0.93		
ACO 25		0.89		

*CFA, confirmatory factor analysis; ACO, attitudes toward communication; CR, composite reliability; AVE, average variance extracted.*

### Attitudes Toward the Communication of Nursing Students With the Patient, Relationships Between the Academic Course, and Correlations Between Dimensions of Attitudes Toward Communication Instrument for Nursing Students

Analysis of attitudes toward patient communication of nursing students provided the following results: behavioral (M = 4.56; *SD* = 0.69) and cognitive (M = 4.73; *SD* = 0.47) showed the highest scores, meanwhile the dimension related to affective communication (M = 1.65; *SD* = 0.77) had the lowest average score.

Then, we analyzed the relationships between the academic course and the ACO dimensions by using the ANOVA analyses with the Bonferroni *post hoc* test to determine the differences between the variables.

In relation to the academic course (first, second, third, and fourth courses), the results of the ANOVA statistically significant differences in all the dimensions of the ACO instrument for nursing students were observed (Table 3). Statistically significant differences in the Bonferroni *post hoc* tests in the affective dimension were reported between the second and the other courses, with higher differences in the second-year students. With respect to the cognitive and behavioral dimensions, statistically significant differences were shown between first-, second-, and fourth-year students, with the highest values in first-year students.

**TABLE 3:** Items of the ACO scale adapted for nursing students according to the academic course.

ACO dimensions	First (SD)	Second (SD)	Third (SD)	Fourth (SD)	F	*p*-value
Affective	1.56 (0.54)	1.82 (0.82)	1.55 (0.57)	1.65 (0.68)	12.8	0.000***
Cognitive	4.85 (0.47)	4.69 (0.75)	4.77 (0.58)	4.70 (0.74)	4.8	0.000***
Behavioral	4.66 (0.53)	4.49 (0.76)	4.57 (0.66)	4.39 (0.82)	9.2	0.003**

*p ≤ 0.05; **p ≤ 0.01; ***p ≤ 0.001.

Finally, correlations between the different dimensions of the ACO instrument for nursing students were tested. In the case of the affective dimension, statistically significant, negative and low correlations were observed with the behavioral (*r* = −0.32; *p ≤ 0.05) and cognitive (*r* = −0.36; *p ≤ 0.05) dimensions. The behavioral and cognitive dimensions showed a significant, positive, and very high correlation (*r* = 0.90; *p ≤ 0.05).

## Discussion

This study showed the adaptation and validation of the instrument to measure the ACO of nursing students. Analysis of the 25 items demonstrated an adequate contribution to the overall scale. The reliability of the construct showed an acceptable coefficient (Cronbach’s α = 0.84) above the minimum value (>0.70) indicated in the literature (Nunnally, 1978) and did not appear to be improved by removing any of the items. As for the construct validity of the scale, the EFA results showed that the 25 items of the variance explained by the three factors was 78%, RMSR value of 0.03 (<0.50; Harman, 1980), and a GFI index of 0.99 (>0.95; Tanaka and Huba, 1989). This indicated an adequate fit, CFA also replicated that structure, and presented an adequate fit (χ^2^ = 2347.59; df = 272; *p* < 0.05; NNFI = 0.91; CFI = 0.92; IFI = 0.92; and RMSEA = 0.07). The RMSEA was 0.07, which agrees with the optimal values fit criteria (≤ 0.08) (Browne and Cudeck, 1993). Similarly, the remaining indices showed a good fit: NNFI = 0.91; CFI = 0.92; and IFI = 0.92 (values over 0.90 indicate an adequate fit) (McCallum and Austin, 2000). Overall, the obtained psychometric properties suggest that the instrument is reliable and valid, which justifies its use to assess ACO in nursing students. These results are relevant because communication is a core competence in nursing students (Chang and Chang, 2021) and behaviors can be predicted by studying attitudes. Then, assessing the ACO for nursing students with the patient is critical as future nursing professionals. Studying the development and modification of attitudes involve exposure to new information (theory lessons, the importance of communication, benefits, and communicative moments), imposed behavioral change (experiential lessons, communication behavioral, and role play), and an increase in self-knowledge (answering an instrument increasing the awareness of the students of their attitudes). Increased knowledge and awareness of their attitudes and behaviors may have already contributed to changing attitudes in nursing students (Koponen et al., 2012). Despite the importance of attitudes in predicting behaviors, no studies have been found assessing ACO in nursing students (MacLean et al., 2017; Levett-Jones et al., 2019), but on communication skills, communication knowledge, and in medical students (Epstein et al., 2010; Škodová et al., 2018; Givron and Desseilles, 2021). Therefore, it is essential to have instruments with adequate psychometric properties to be aware of the attitudes in order to improve negative attitudes and contribute to the development of training programs adapted to the real needs of nursing students.

With respect to the attitudes of nursing students, it seems to indicate positive ACO with the patient. The dimensions with the highest scores were cognitive and behavioral, while scores were worst in the affective component. The affective component showed an inverted dimension when subjects were asked about communication anxiety. These results were in the same line as the Giménez-Espert and Prado-Gascó (2018) study carried out with a sample of the nurse. This can be explained by the fact that the three attitude components are related, since the experienced feeling (emotional dimension) is mainly based on knowledge (cognitive dimension) and actions are guided by feelings and by knowledge (behavioral dimension) (Fishbein and Cappella, 2006; Ertz et al., 2016).

According to the academic course of nursing students, the ANOVA statistically significant differences in all the dimensions of instrument ACO were found. The second-year nursing students showed more positive attitudes in the affective dimension, while in the cognitive and behavioral dimension, the most positive attitudes were found in the first-year students. These findings are consistent with previous studies, showing that communicative attitudes become more negative over time because students may be exposed to more negative communicative experiences, as they get older and may also experience difficulties with increasingly demanding communicative situations (Clark et al., 2012). Due to the curriculum demands and time constraints, it leads to prioritizing clinical skills over interpersonal skills (Cusatis et al., 2020). In addition, the upper class nursing students have already had experiences with the professional world during their clinical practice, which implies exposure to communicate complex situations, human suffering, without adequate educational preparation and support (Ward et al., 2012).

Finally, in the correlations, the behavioral and cognitive dimensions showed a significant, positive, and very high correlation, according to the literature (Rosenberg and Hovland, 1960; Ajzen, 1991). Communication is essential for the quality of care and satisfaction of the patient (Finke et al., 2008; Kourkouta and Papathanasiou, 2014; Finney Rutten et al., 2015; Banerjee et al., 2016; Gillett et al., 2016; Howick et al., 2018) and for the effectiveness of healthcare teams, it can be related to the quality of care and job satisfaction of healthcare workers, such as nurses (Gausvik et al., 2015). Moreover, the influence of knowledge and attitudes on communication is evident (Mullan and Kothe, 2010); therefore, it is necessary to deepen the study of the ACO of nursing students.

In spite of the advantages of this study, several limitations were present. Nevertheless, because the sampling was not probabilistic and the subjects were exclusively from Valencian Community, the results should be generalized with caution. A large sample of nursing students was used in this study. Future studies would be interesting to extend this study to other populations in Spain and in other Spanish-speaking countries. Another limitation is related to the use of self-reports to collect data, and they can introduce bias through the phenomenon of social desirability (Rammstedt et al., 2017). In future research, it would be advisable to combine the results on another type of instrument completed by others and/or with external objective measures, perform a comparison to another measure gold standard, and potential outcome to nursing students’ attitude to communication with patients.

Hence, the importance of analyzing the ACO in nursing students generates a need for reliable and validated instruments. In conclusion, the existence of appropriate instruments allows the measurement of the ACO, evaluating educational needs, developing interventions adapted to real needs, and assessing the interventions developed to improve the ACO in nursing students. These findings should be considered in developing academic plans to improve the effectiveness of the communication education process of the students to increase the quality of patient care and well-being of the nursing students.

## Data Availability Statement

The raw data supporting the conclusions of this article will be made available by the authors, without undue reservation.

## Ethics Statement

The studies involving human participants were reviewed and approved by the Human Research Ethics Committee of the University of Valencia H1529396558647. The patients/participants provided their written informed consent to participate in this study.

## Author Contributions

MCG-E, SM, DP, and VP-G made a substantial contribution to the concept, design of the work, acquisition, analysis, and interpretation of data, drafted the manuscript and revised it critically for important intellectual content, and participated sufficiently in the work to take public responsibility for appropriate portions of the content. All authors contributed to the manuscript and approved the submitted version.

## Conflict of Interest

The authors declare that the research was conducted in the absence of any commercial or financial relationships that could be construed as a potential conflict of interest.

## Publisher’s Note

All claims expressed in this article are solely those of the authors and do not necessarily represent those of their affiliated organizations, or those of the publisher, the editors and the reviewers. Any product that may be evaluated in this article, or claim that may be made by its manufacturer, is not guaranteed or endorsed by the publisher.
